# The 3T model of military veteran radicalization and extremism: exploring risk factors and protective strategies

**DOI:** 10.3389/fsoc.2025.1500774

**Published:** 2025-03-12

**Authors:** Hazel R. Atuel, Carl A. Castro

**Affiliations:** Center for Innovation and Research on Veterans & Military Families, University of Southern California, Los Angeles, CA, United States

**Keywords:** radicalization, violent extremism, military veterans, risk factors, protective strategies

## Abstract

In the aftermath of several wars within the last century, seminal research forewarned against the rising tide of radicalization and violent extremism (VE) among military veterans. Building on the pioneering work, the current study explores risk and protective factors related to military veteran extremism. Utilizing the retrospective thick description approach, the study utilized both primary (e.g., interviews) and open-source (e.g., court transcripts) data to examine and contextualize the VE trajectory across the military lifecycle (premilitary, military, postmilitary), as informed by people from various social networks (e.g., family, civilian/premilitary). The select sample comprised 30 VE veterans and 30 VE civilians who committed/planned a VE act between 2003 and 2019, and a comparison group of 10 non-VE veterans (i.e., veterans who resisted radicalization and VE). Directed content analyses results yielded a conceptual model reflecting three general risk factors (*Transmission of Prejudice, Trauma and Adversity, and Transition*) common among civilian and veterans alike. In addition, behavioral and cognitive strategies related to three general protective strategies (*Resistance against Transmission of Prejudice, Addressing Trauma and Overcoming Adversity, Navigating Transitions*) were found to steer veterans away from radicalization and VE across the military lifecycle. Implications for future research are discussed.

## Introduction

*“The country does not know it yet, but it has created a monster, a monster in the form of millions of men who have been taught to deal and to trade in violence….men who have returned with a sense of anger and a sense of betrayal which no one has yet grasped….”*

-Kerry and Vietnam Veterans Against the War (1971)

The argument can be made that the January 6, 2021 insurrection (J6) was bound to happen. More than 70 years ago, against the backdrop of the Holocaust, five research projects were commissioned to understand the individual dynamics of prejudice: *The Authoritarian Personality* (Adorno et al., 1950) explored personality traits to illuminate conformity to the point of violence; *Dynamics of Prejudice: A Psychological and Sociological Study of Veterans* (Bettelheim and Janowitz, 1950) examined the wartime experiences of former servicemembers; *Anti-Semitism and Emotional Disorder* (Ackerman and Jahoda, 1950) investigated the clinical correlates of anti-Semitism; *Rehearsal for Destruction* (Massing, 1949) provided the historical progression of Nazi anti-Semitism that was used as propaganda to gain political and economic advantage; and *Prophets of Deceit: A Study of the Techniques of the American Agitator* (Lowenthal and Guterman, 1949) analyzed the persuasion techniques used by authoritarians to manipulate the masses. While each study provided unique insights, a holistic understanding of prejudice leading up to mass violence required an integration of the findings (Horkheimer and Flowerman, 1950).

This set of studies was intended to advance a research agenda that provided practical solutions to prevent the atrocities of the past. Because almost all of the U.S.-based social scientists (i.e., psychologists, sociologists) who led these efforts were émigrés who fled Nazi Germany, they saw similar conditions brewing in their adoptive country as World War II drew to a close. It was only a matter of time when these individual-level factors came together and conspired, this time, to threaten American democracy in the 21st century.

While J6 has generated significant interest in radicalization and violent extremism (VE) among people with a military background (hereafter referred to as veterans), this research area is still in its infancy. However, *prior to J6*, seminal veteran studies provided insights that could explain the radicalization process and, by and large, forewarned the rise of VE among veterans. The present study is situated within this literature and builds on the early work as well as a few contemporary studies, to which we briefly review.

## Radicalization and violent extremism among military veterans

### Pioneering work

#### Civil War and World War I

Waller (1944), a World War I veteran, pointed out the pattern of experiences and grievances that fueled ‘counterrevolutions’ among veterans of early wars (pp. 6–16). In some cases, these rebellions are modern-day equivalents of terrorism, as exemplified by the rise of the Ku Klux Klan in the aftermath of the Civil War. Broadly, veterans’ sense of bitterness against institutions (e.g., government) arose from failure to secure basic (e.g., employment) and psychological (e.g., persistence of military identity) needs. In turn, these grievances culminated in seeking like-minded others, with the goal of disrupting the status quo even through violent means. Well aware of the variance within the veteran population with the majority being able to reintegrate successfully, Waller (1944) advocated for an interdisciplinary approach to veteran studies that includes examining the interactive effects of civilian temperament (premilitary), military service, and veteran experience (postmilitary). Written during the rise of fascism in Europe in the last century, Waller (1944) cautioned that veterans are a ready tool for a demagogue (p. 188) who will capitalize on veteran grievances and offer to provide solutions to their problems.

#### World War II

Toward the close of World War II, Bettelheim and Janowitz (1950) examined the wartime experiences, and the anti-Semitic and anti-black people/persons/person attitudes of a sample of U.S. veterans. The focus on a veteran sample was based on historical accounts that World War I German veterans who faced civilian transition challenges were the “chief promoters and followers of the anti-Semitic movement” (p. 4) and “had a strong desire to see violent change in the structure of a society which they felt had let them down” (p. 5). More than 70 years ago, the study authors were aware that preventing Nazism from taking root on U.S. soil required assessing the attitudes and situations of veterans during their civilian transition.

The sample comprised 150 Army veterans, all white males, discharged from service between 6 and 8 months previously, living in metropolitan Chicago, and interviewed for almost 8 h using an open-ended questionnaire. Based on participant responses, the sample was categorized into four groups: *Tolerant* (i.e., endorsed few stereotypes of Jews [41%] and black people/persons/person [8%]), *Stereotyped* (i.e., expressed various stereotypes of Jews [28%] and black people/persons/person [27%]), *Outspoken* (i.e., held unfavorable stereotypes and expressed hostility against Jews [27%] and black people/persons/person [49%]), and *Intense* (i.e., exhibited spontaneous hostility against Jews [4%] and black people/persons/person [16%]). It is noteworthy that while everyone in the sample demonstrated outgroup stereotyping, only some expressed outgroup hostility.

Of relevance are the findings for the Outspoken and Intense subgroups: perceived future economic deprivation and downward social mobility were associated with greater intolerance of Jews and black people/persons/person. A look at the qualitative results revealed the reference point for downward social mobility was the premilitary/civilian status. The authors explained these subgroups of veterans felt their military service justified the expectation of better employment opportunities, and not receiving special treatment for their military service was perceived as a mistreatment by society. Specifically, Jews and black people/persons/person were perceived as threats to the veteran’s own economic advancement.

Other notable findings revealed that the Outspoken and Intense subgroups (1) held stereotypes of Jews and black people/persons/person *prior* to their military service, (2) had a history of poor adjustment in civilian society prior to military service, (3) avoided reality testing by adopting stereotypic thinking through the acceptance of conspiracy theories, (4) held anti-government beliefs, (5) felt the government was not doing enough for veterans, and (6) felt disconnected from the broader society.

#### Vietnam War

Retzer (1976), a Vietnam War Veteran, conducted an in-depth examination of the radicalization of his veteran peers. The findings revealed that while all participants were not radicals prior to military service, their post-war experience equally divided them between non-radicals and radicals. Among radical veterans, a pattern of results emerged for the premilitary and military phases. Prior to military service, the radical veterans reported a growing sense of community alienation. To cope, they challenged community norms and practices based on alternative principles or values. In other words, they were already on the fringe and learned to navigate life from this marginalized standpoint. During their military service, they found themselves betrayed by their leaders and appalled at their own complicity as executioners of amoral orders (p. 355). Hence, the war experience was the impetus for engaging in previously learned attitudes and behaviors (from the premilitary phase). Radicalization among veterans, then, appears to be a product of premilitary norms and values interacting with military combat experiences, values, and norms.

Briefly, what could have been a robust multidisciplinary field (see Waller, 1944) seemed dormant for decades. We raise this issue because in 2009, the Office of Intelligence and Analysis (2009) similarly forewarned that veterans experiencing civilian reintegration difficulties are fertile ground for extremist ideology to flourish. In the interim, few studies were conducted, in part, to address more recent historical VE events.

### Contemporary studies: resurrecting the military experience

In the aftermath of the 1995 Oklahoma City Bombing led by Timothy McVeigh, an Army veteran, and the 1995 Fayetteville murders of a Black man and woman by three white servicemembers, Curtin (1997) conducted one of the few studies on white nationalist extremism among servicemembers. He observed certain common demographics among servicemembers who joined these groups. These included being a young adult (18–25) and living in impoverished circumstances during childhood years, or being middle-aged and having a middle-class lifestyle. When mapped onto the military career trajectory, these results suggest that those who are in the early stages or transitioning into or in the later stages or transitioning out of military service are at-risk of joining extremist groups.

A closer look at exit from military service, Simi et al. (2013) used sociological frameworks to explain the relationship between military service and far-right violence. Results from their case study approach suggest that exit from the military appears to be an initial pathway to far-right extremism leading up to violence. Among Vietnam War veterans who voluntarily exited from the military, the unwelcome climate of civilian society made the transition experience a difficult one, creating social stress (e.g., anger), which was a gateway for recruitment into far-right extremist groups. On the other hand, veterans who involuntarily exited from the military (e.g., failure to advance in rank), experienced identity incongruence (i.e., they saw themselves as warriors rejected by the military), which motivated them to seek extremist groups with a para-military arm to reinforce their warrior identity.

Finally, in a review of findings from various studies funded by the National Institute of Justice’s Domestic Radicalization to Terrorism program, Smith (2018) observed that people who commit violence, in general, shared potential risk factors with people who engaged in domestic terrorism. Common risk factors include having a history of criminal violence, having a criminal history, having military experience, having psychological issues, being unemployed, failing to achieve one’s aspirations, and being male. Moving forward, one recommendation was for future studies to combine secondary analyses of available data and conduct primary data collection (e.g., interviews) to gain deeper insights for the development of risk assessments.

### Summary

The early sociological and psychological work among Civil War and World War I (Waller, 1944), World War II (Bettelheim and Janowitz, 1950), and Vietnam War (Retzer, 1976) veterans laid the foundation for understanding and identifying factors that put veterans at-risk for radicalization leading up to VE. Foremost, the radicalization process appears to follow a trajectory similar to the military lifecycle, with initial exposure to radical narratives and beliefs occurring before military service, during military service, and after military service. In addition, military life experiences, especially related to war, coupled with postmilitary transition difficulties (e.g., basic needs) can inform veterans’ grievances to associate with radical groups. Meanwhile, contemporary sociological and psychological studies have added to the empirical base by identifying entry into and exit from the military as critical transition timepoints (Curtin, 1997), identity incongruence among veterans as a motivating factor for radicalization (Simi et al., 2013), as well as psychological vulnerabilities and criminal propensities (Smith, 2018).

Taking these findings as directives, we pose the following questions: Who introduced veterans to extremist ideas? Their family, civilian friends, military comrades, or veteran peers? Do veterans who engage in VE share similar risk factors as civilians who engage in VE? Given the tempo of military and postmilitary life, what other military experiences (i.e., beyond war and in between entry and exit into military service) and other postmilitary experiences (i.e., beyond transition challenges and identity incongruence) could be related to the radicalization of veterans?

## The present study: the military radicalization (MRad) project

Broadly, the present study comparatively explored veterans and civilians who engaged in VE, allowing for an examination of similarities and differences in risk factors between VE veterans and VE civilians. Given that VE is considered a “low frequency, high impact” event means that the majority of veterans have not engaged in VE. Simply put, VE is an exception, not the norm among veterans. Hence, the project also explored similarities and differences in risk factors between VE veterans and non-VE veterans, and protective factors among non-VE veterans.

### Theoretical frameworks

We borrow from several lines of theory and research that have a direct bearing on the current study including the Quest for Significance Theory (Kruglanski and Fishman, 2009; Kruglanski et al., 2013, 2014, 2018; Webber et al., 2017, 2018), the military lifecycle depicted in the Military Transition Theory (Castro and Kintzle, 2018), research on the social networks within the military lifecycle (Atuel et al., 2016), and Veteran Identity Theory (Atuel and Castro, 2018a, 2018b).

#### Quest for significance

Based on decades of research with both ideological and violent extremists, Kruglanski et al. (2013, 2014) developed a psychological theory of VE that primarily takes into account an individual’s motivation to satisfy a dominant need and ability to carry out violent activities. The theory holds that people have varying types of needs (e.g., basic, social) and strive to fulfill those needs within the constraints of the mainstream. When a particular need becomes dominant (e.g., hunger), it may compel people to engage in deviant behavior (e.g., stealing) where normative behavior that would satisfy that need appears to be unavailable.

In the case of VE, the dominant need pertains to a loss of worth, esteem, or meaning which, owing to our social nature, has negative implications for people’s self-concept or social standing within the mainstream. When people are consumed by this dominant need, all other needs become inhibited (Kruglanski et al., 2009, 2013, 2014). As a consequence, the constraints those needs exercise on behavior are removed or significantly weakened. This permits extreme behaviors to be enacted that formerly were constrained and hence prohibited.

Obviously, the quest for significance is not a sufficient condition for violence. In addition, people should subscribe to an ideological *narrative* that portrays violence as the path to significance. Typically, too, the violence-significance link must be supported by a social *network*—such as the one formed by McVeigh, Nichols, Fortier during basic training in the Army—that validates the narrative and rewards those who implement it in action (by practicing violence against targets identified in the narrative). Within the radical network, VE is reinforced and even rewarded, thereby setting a standard for members to follow or emulate. What mainstream considers as deviant, the radical network deems as normative.

In sum, the quest for significance leading up to VE reflects an individual who has the ability to carry out a violent act that is justified by an extreme ideology and supported by a radical network. While this theory has been used to explain VE among the civilian population, it has yet to be applied to the veteran population, particularly to the military lifecycle, to which we now turn.

#### Military lifecycle

The Military Transition Theory (Castro and Kintzle, 2018) aims to describe and predict important aspects of transitions that occur throughout a servicemember’s life (premilitary/civilian, military, postmilitary/veteran).

Premilitary refers to the civilian life prior to enlistment in military service (Brookover, 1945; Hollingshead, 1946). During the premilitary phase, people have a civilian social network reflecting familial ties and friendships that provide informational (e.g., advice), emotional (e.g., safety), and instrumental (e.g., finances) supports (Beardslee et al., 2013; Wadsworth et al., 2013). Because most recruits are in their late teens (Bachman et al., 2000), their close relationships are tied to their school, religious organization, or various community organizations.

When civilians enlist into the military service, they enter the military phase, which is characterized by a series of indoctrination and trainings (e.g., basic, combat) aimed at preparing them for war (Castro and Hassan, 2023). It is during this period that civilians are transformed into warriors, an identity that is unique to the military, eventually setting them apart from their civilian peers. Three factors interact to define the boundaries delineating military and civilian cultures, and provide critical information on the military social network and military identity.

##### Chain of command structure

The organizational structure of the military is comprised of a power hierarchy known as the chain of command (Brotz and Wilson, 1946) that revolves around a succession of commanding officers differentiating superior and subordinate roles. This power hierarchy is critical in identifying servicemembers’ rightful place and dictating appropriate behavior based on servicemembers’ role and status. In other words, the chain of command is a social network of interdependent roles within an ordered power hierarchy.

##### Military norms

As a cultural group, the military has its own history and norms (Atuel and Castro, 2018a). Military norms encompass the spectrum of beliefs, values, traditions, behaviors, and events directly related to military service and life, as well as the language used in communicating within the chain of command. Simultaneously, servicemembers learn the values of honor, integrity, commitment, loyalty, respect, and devotion to duty.

##### Military identity

The military is often described as a “warrior culture,” whereby servicemembers are in a constant physical and psychological state of “combat readiness” (Castro and Adler, 1999). Unlike most civilian jobs, military service is a 24-h, 7-day occupation. Additionally, even while not in uniform, servicemembers are expected to uphold military laws (e.g., Uniform Code of Military Justice), norms, and rules of conduct (Coll et al., 2012).

Ultimately, military service comes to an end, ushering the postmilitary phase. Veteran status means withdrawal of military protection and support (with the exception of veteran benefits), and providing for one’s self in the civilian setting. Exit from the military means fending for oneself in the civilian world. As veterans, their premilitary social network (e.g., civilian friends) regains importance and, typically, they acquire a veteran social network through their receipt of veteran benefits or affiliation with community-based veteran groups. At times, veteran social networks have been found to be a greater source of information and emotional support during the civilian reintegration process (e.g., Demers, 2011).

##### Veteran identity

Emerging evidence has uncovered the day-to-day struggles some veterans face in their civilian transition including employment, housing, finances, and access to health care (Castro et al., 2014, 2015; Castro and Kintzle, 2017). Moreover, navigating through the difficulties of transitioning back into civilian life is compounded by a deeper struggle pertaining to the perseverance of the military identity in the civilian context (Atuel and Castro, 2018b; Atuel et al., 2016). Early research has identified this as one of the root causes of difficulties in the civilian transition (Hollingshead, 1946). Some can even be categorized as dogmatic veterans/reluctant civilians (Atuel and Castro, 2018b). This occurs when veterans have not yet formed or failed to form a civilian identity that is a source of self-esteem similar to that of the military identity. A special case that would fall into this category as well is when veterans have formed a civilian identity, but found it to be relatively insignificant as a source of self-esteem compared to the military identity. In all these instances, this subgroup of dogmatic veterans or reluctant civilians psychologically “stay” in the military group.

#### Quest for significance: implications for the military lifecycle and veteran identity

Significance Quest Theory interfaces with that of the military lifecycle in addressing the varying conditions and possibilities of fulfilling the significance need across the various phases. Presumably, joining the military is motivated by significance need, either through the ideal of serving one’s country, acquiring the status of warrior/hero, and/or through the educational and training opportunities the military offers (e.g., Miller, 2010; Ngaruiya et al., 2014). The military identity initially acquired at training and strengthened during service is a highly positive one and is a great source of esteem and pride (Hall, 2012; Lancaster and Hart, 2015). The military network, as a whole, subscribe to the overarching narrative that military service is honorable, prestigious and widely respected, thus augmenting servicemembers’ self-worth and significance.

Servicemembers’ sense of personal significance may change once they leave active duty. As veterans, people may part ways with the cohesive social network of military camaraderie, consequentially lowering veterans’ sense of personal significance in comparison to its level during active service. If in addition veterans may encounter difficulties in finding significance lending employment, they might be susceptible to radicalizing narratives that advocate violence against the government or other targets as a path to significance.

### Research questions

Hence, we posed two overarching research questions:

In comparing civilians and veterans’ trajectory toward VE, what were the needs, narratives, and networks that put them at-risk for violence, as informed by people from their various social networks (i.e., premilitary, military, postmilitary)? To strengthen the research design, a group of veterans who have not engaged in VE (non-VE veteran group) served as a comparison group. Second, among non-VE veterans, what were the needs, narratives, and networks that made them resist radicalization and VE across the military lifecycle?

## Method

The study utilized a retrospective thick description approach (Geertz, 2008) to examine the VE pathway among civilians and veterans. In doing so, we are able to contextualize the trajectory from “biographical, historical, situational, relational, and interactional” (Denzin, 2001, p. 10) perspectives, thereby allowing for an in-depth examination of risk factors across an individual’s life history. Moreover, this method advocates for triangulated and multiple sources of information that can reflect the social networks of people as they change across time.

### Samples and procedure

#### Institutional review board

The study was approved by the University of Southern California Institutional Review Board (UP-20-00969).

#### VE civilian, VE veteran, and non-VE veteran samples

##### VE civilian and VE veteran samples

Prior to obtaining the social network informant sample, we decided to focus the study on a select sample of civilians and veterans who enacted/planned a VE act between 2003 and 2019. One justification for the timeframe and the limited number in the sample is to provide continuity to a previous study (see Atuel and Castro, 2024) that examined civilians and veterans who were indicted for domestic terrorism by the U.S. government between 1980 and 2002. Second, a sample size of 30 civilians and 30 veterans falls within the acceptable range (between 25 and 30) to reach saturation and redundancy in qualitative research (Dworkin, 2012). Admittedly, other civilians and veterans can be included in the list, but for purposes of this exploratory study, the sample is limited to 30 in each group.

Briefly, far-right ideologies (i.e., White Supremacy, Anti-Government) were predominant among VE civilians (81%) and VE veterans (70%), followed by Radical Islam (20% for VE civilians and VE veterans). Meanwhile “Black” nationalism among VE veterans (10%) could be a historical artifact given the VE veterans in our sample committed their act in retaliation against high-profile killings of black people/person/persons between 2003 and 2019.

##### Non-VE veteran sample

Veterans exposed to and resisted radical ideologies (e.g., White Supremacy, Anti-Government) served as a comparison group. This sample was recruited from a combination of snowball sampling (i.e., one veteran knew of another veteran who was exposed to and resisted violent ideology), outreach to various community-based veteran organizations (e.g., Veterans Village), and social media outreach to active-duty and veteran forums.

Non-VE veterans provided informed consent for the virtual interview using the Zoom platform, and notified that interviews will be audiotaped and transcribed for analysis. Interviews lasted between 60 and 180 min and participants were provided with a $50 e-giftcard stipend at the end of the interview.

#### Social network informants

The sample comprised persons situated within the respective networks of each VE civilian/veteran. Networks contained individuals (e.g., friends or classmates for the premilitary network) who provided information about the VE civilian/veteran through open-source data (e.g., court transcripts, media outlets) or by participating in an online interview conducted by the study team.

Social network informants who initially provided information through open-source data were contacted and recruited to participate in the study. Participants provided informed consent for the virtual interview using the Zoom platform, and notified interviews will be audiotaped and transcribed for analysis. Interviews lasted between 60 and 90 min and participants were provided with a $50 e-giftcard stipend at the end of the interview.

#### Final informant sample

There were 92 family informants, 108 civilian/premilitary informants, 64 military informants, and 31 postmilitary informants (see Table 1). Of the 30 VE civilians and 30 VE veterans, 25 VE civilians and 26 VE veterans self-disclosed through writings (e.g., screeds, self-published books) or interviews (e.g., investigative journalism). As mentioned, 10 non-VE veterans served as the comparison group.

**Table 1:** Types and frequency of social network informants for VE civilians (*N* = 30), VE veterans (*N* = 30) and non-VE veteran (*N* = 10).

Type of social network	VE civilian	VE veteran^*^	Non-VE veteran
Self^a^	25	26	10
Family^b^^,^^g^	48	43	1
Civilian/Premilitary^c^^,^^g^	76	32	0
Military^d^^,^^g^	N/A	64	0
Postmilitary^e^^,^^g^	N/A	31	0
Other^f^^,^^g^	44	29	0

a Self data derived from interviews or writings (e.g., self-published books, manifestos, screed, suicide notes).

b Family refers to parents, (ex-)spouses, children, and other relatives.

c Civilian/Premilitary refers to friends, neighbors, classmates, or teachers in a civilian setting/prior to military service.

d Military refers to peers, supervisors, friends, or neighbors during military service.

e Postmilitary refers to friends, neighbors, supervisors, peers after military service.

f Other refers to attorney statements, clinical evaluations, unclassified FBI/LE report.

g Data derived from interviews, court exhibits/transcripts or interviews given to various media outlets.

* Per one of the reviewers, the informant sample for the VE veteran group should be noted as referring “to their highly subjective assessments of family relationships, childhood, school years, aspects of their military career and post-military issues, which could be distorted by memory errors”.

### Measures

#### Interview data

For the VE civilian/veteran samples, semi-structured interviews were developed for the family, premilitary, military, and postmilitary informants. Broadly, the interview contained questions that asked the informant to recall their knowledge of the VE civilian/veteran’s premilitary life (e.g., family relationships, childhood, school years, aspirations, motivations), military life (e.g., specific duties, achievements, getting in trouble), postmilitary life (e.g., educational experience, employment experience, grievances), and radicalization and VE engagement (e.g., exposure to radical/extremist beliefs or individuals, content of radical/extremist beliefs).

Another set of semi-structured interviews were developed for the non-VE veteran sample and their social network. Generally, similar questions were asked of the non-VE veteran and their social network informants in terms of the non-VE veteran’s premilitary life (e.g., family relationships), military life (e.g., getting in trouble), and postmilitary life (e.g., employment experience). Additional questions for the non-VE veteran pertained to exposure to and resistance to extremist beliefs (e.g., content of radical or extremist beliefs, cognitive and behavioral resistance strategies).

#### Open-source data

The study team also compiled data from open sources including court documents (e.g., transcripts, exhibits), media outlets (e.g., investigative journalism, interviews), peer-reviewed articles in academic journals, reports released by established organizations (e.g., Southern Poverty Law Center, Anti-Defamation League), unclassified government/law enforcement reports, and self-published books or writings.

### Data analytic strategy

After culling through all available interview and open-source data, the research team utilized directed content analysis (DCA; Hsieh and Shannon, 2005) to analyze the data. Using DCA allowed for the use of *a priori* coding categories (i.e., needs, narratives, networks) and the creation of new coding categories and sub-categories during the analysis process. The analyses were conducted in two stages, with the first stage focused on analyzing data within a phase (e.g., premilitary/civilian), and the second stage directed toward analyzing data across transition phases (e.g., from military to postmilitary).

The overall coding process was iterative. First, each VE civilian/veteran and non-VE veteran file that contained all interview and open-source data was coded by 3 independent raters that included one of the PIs. Regular research meetings involving all raters and the 2 study PIs were held to discuss emergent findings. Second, the PIs held several meetings directed toward finalizing the categories/subcategories within and between transition phases, bringing in raw data when necessary for further exploration and clarification, to finalize the categories/subcategories.

We provide examples of the various coding processes in Tables 2–4, with each table reflecting raw data excerpts, initial coding excerpts, and final category and subcategory excerpts for each person case. Table 2 reflects transmission of prejudice coding within the premilitary phase across the three subgroups. Meanwhile, Table 3 reflects the transmission of prejudice coding across the military lifecycle for a VE veteran. Lastly, Table 4 reflects resisting transmission of prejudice coding across the military lifecycle for a non-VE veteran.

**Table 2:** Coding examples of transmission of prejudice within the premilitary/civilian phase (*n* = 1 VE civilian, 1 VE veteran, 1 non-VE veteran).

	Raw data excerpts	Initial coding excerpts	Final category and subcategory excerpts
VE civilian	“… [VE civilian] *had already written to the court to say that [their] racist views had formed during [their] childhood as mother frequently used hateful racist language at home*” “… [VE civilian’s mother] *admitted she had used the racial terms* [their child] *had outlined in* [their] *letter. She apologized for lying and for the miseducation of* [child]. *She stated that her racial views had been planted by her family during her formative years.”*	- Network: Family - Narrative: Hateful, racist language	Category: Transmission of Prejudice Subcategory: Exposure through Family
VE veteran	“…[parent’s name] *was highly influential in forming* [VE Veteran’s name] *antigovernment views*.”	- Network: Family - Narrative: Antigovernment	Category: Transmission of Prejudice Subcategory: Exposure through Family
Non-VE veteran	“…[father] was still prejudiced. He still said n**** all the time. That was just a word he grew up and he used it commonly.”	- Network: Family - Narrative: Prejudice; racist language	Category: Transmission of Prejudice Subcategory: Exposure through Family

**Table 3:** Coding examples of transmission of prejudice of a VE veteran across the military lifecycle.

	Raw data excerpts	Initial coding excerpts	Final category and subcategory excerpts
Premilitary/civilian phase	“*Uncle* [name] *died before I got involved in White racist groups, but he and I shared the same racial and political views, and spoke together for hours on end during my weekend visits…I came to know him well while I was growing up…”*	- Network: Family	Category: Transmission of Prejudice Subcategory: Exposure through Family
Military phase	*““Hitler was a White racist and so was I. And, like him, I wanted to unite, organize, and educate the White masses…. In many ways, I would try to emulate Hitler’s methods of attracting members and supporters…. It had been successful with Hitler, and I felt it would be successful with me.”* *“Also joining…were several dozen active duty Marines from* [name of installation]*…… and we had three active Dens in that area….”*	- Network: Military	Category: Transmission of Prejudice Subcategory: Exposure through Military
Postmilitary phase	“[Name of U.S. Army veteran]….*first visited me… and after several hours of feeling me out and satisfying himself that our political and racial views were compatible joined….”*	- Network: Veteran	Category: Transmission of Prejudice Subcategory: Exposure through Veteran

**Table 4:** Coding examples of resisting transmission of prejudice of a non-VE veteran across the military lifecyle.

	Raw data excerpts	Initial coding excerpts	Final category and subcategory excerpts
Premilitary/civilian phase	“*Both my parents were anti-fascists and I still am…”* *“I was a reader….Science fiction. I read science fiction, which is basically a lot of, it’s sociology, which is a real interest of mine, and some politics….My favorite book when I was a kid was A Wrinkle in Time. It was on the sixth grade shelf and I read it in fourth grade….You read books in your head.”*	- Network: Family (Note: Father was a WWII veteran) - Narrative: Anti-fascist - Narrative: Science fiction, sociology, politics	Category: Moral Foundation Subcategory: Family values; family resistance Subcategory: Reading as a habit
Military phase	*“…I went to* [name of military] *academy….the guy that was doing the racist crap….I told him, I said, stop that. It’s nasty. It’s not nice. Please do not do that. It offends me. That did not stop him. That was pouring gas on a fire for that guy.”*	- Network: Military - Narrative: Racist language - Response: Speak up against racism	Category: Moral Foundation Subcategory: Courage (doing the right thing)
Postmilitary phase	*“…from Oathkeepers. It was a piece of paper and it was like upholding your oath that you took and I thought about that….but there’s no ETS on my uphold and defend the constitution thing I said….The next time I heard about* [Oathkeepers]*, it was like, no, I’m staying away from these guys. I dunno what happened to piece of paper. I probably recycled it long ago. I’ve managed to avoid getting suckered into those groups and that’s the way I see it as getting suckered in. Because you do not have the ability to think rationally and say, wait a minute.”*	- Network: Veteran - Narrative: Oathkeepers, antigovernment - Response: Think rationally, avoid group	Category: Moral Foundation Subcategory: Justice (knowing the right thing); Temperance (avoidance of extremes)

The final qualitative coding underwent further reduction and transformed into quantitative variables that are part of the datasets submitted to the National Archives of Criminal Justice Data (NACJD_NIJ-194832). For purposes of the current research, we present the final categories and subcategories that informed the risk factors and protective strategies contained within the 3T model of military veteran extremism.

## Results: demographic and military characteristics

### Demographic characteristics

The non-VE veteran group was older than the VE civilian and VE veteran groups (see Table 5). Also, non-Hispanic white males were the majority across all three groups.

**Table 5:** Means (standard deviations) and percentage of select demographic and military characteristics of VE civilians (*N* = 30), VE veterans (*N* = 30) and non-VE veteran (*N* = 10).

Characteristic	VE civilian	VE veteran	Non-VE veteran
Demographic			
Age			
Mean (SD)	30 (13.04)	34 (16.77)	57 (18.75)
Range	17–59	20–88	22–81
Sex/gender			
Male	80%	100%^*^	80%
Female	20%	–	20%
Race/Ethnicity			
White (non-Hispanic)	97%	77%	80%
White, Hispanic	3%	3%	–
Black people/persons	–	13%	20%
Asian/Pacific Islander	–	3%	–
Unknown	–	3%	–
Military			
Status			
Failed basic training	–	10%	–
Active duty	–	27%	20%
Separated from Service	–	63%	80%
Mean age			
At entry	–	20	20
At exit	–	26	29
Means years of service	–	6	10
Deployed	–	53%	50%
Military indiscipline	–	50%	50%
Marital problems	–	27%	20%
Mental illness	–	23%	30%
Alcohol abuse	–	30%	20%
Branch			
Army/National Guard	–	67%	40%
Navy/Coast Guard	–	10%	60%
Marine Corps	–	23%	–

SPSS 28 was used to run descriptive statistics (e.g., frequencies, mean).

* The VE veteran sample is 100% male, which is a limitation of the present study.

### Military characteristics

While the mean age at entry into military service was identical, VE veterans were slightly younger and had fewer years of service compared to non-VE veterans (see Table 5). Also, while both veteran groups had relatively similar percentages of deployment and military indiscipline, slightly more VE veterans had marital problems and alcohol abuse problems compared to non-VE veterans. Finally, the Army and Marine Corps branches are overrepresented among the VE veteran sample while the Navy/Coast Guard are overrepresented among the non-VE veteran sample.

### 3T model of military veteran extremism: risk factors and protective strategies

We discuss results in two sections. The first section will introduce the emergent general constructs as risk factors among VE civilians, VE veterans, and non-VE veterans. Building on the emergent constructs, the second section will examine behaviors and cognitions that served as protective strategies among non-VE veterans.

### 3T model of military veteran extremism risk factors

The overall findings revealed three general constructs—Transmission of Prejudice, Trauma and Adversity, and Transitions—that situate civilians and veterans alike at-risk for radicalization and VE (see Table 6).

**Table 6:** Predisposing/risk factors across the military lifecyle^*^.

Premilitary phase	Military phase	Postmilitary phase
*Transmission of Prejudice* - Prejudice and ideology exposure/formation through family and civilian peers - Membership/affiliation with radical group (e.g., online; in-person)	*Transmission of Prejudice* - Prejudice and ideology exposure/formation through military peers - Membership/affiliation with radical group (e.g., online; in-person)	*Transmission of Prejudice* - Prejudice and ideology exposure/formation through postmilitary peers - Membership/affiliation with radical group (e.g., online; in-person)
*Trauma and Adversity* - Childhood stressor/adverse childhood experience - Juvenile alcohol/drug use	*Trauma and Adversity* - Military-related moral failure experience or trauma that fuels military grievance (e.g., hazing/bullying/discriminated against; deployment; combat; military sexual assault) - Marital problems - Alcohol abuse/illegal substance use	*Trauma and Adversity* - Postmilitary moral failure experience or trauma that fuels postmilitary grievance (e.g., discharge status) - Marital problems - Alcohol abuse/illegal substance use
*Transitions* - School discipline - Juvenile contact with LE	*Transitions* - Military Indiscipline (e.g., ART 15, demotion, extra duty); Perceived rejection (e.g., inability to be promoted)	*Transitions* - Postmilitary transition challenges that fuels postmilitary grievance (unemployment, homelessness)

* Adopted from Table 5 of the Final Research Report for 2019-ZA-CX-0002.

#### Transmission of prejudice

*“…*[VE Civilian] *formed racist views during childhood…had been motivated by own mother, who frequently used racial and hateful language at home….”*

*-*VE Civilian Other Informant

*“Within two minutes of browsing through this 16-page tabloid* [given by father who obtained it from a neighbor]*, I knew I had found a home within the American White Movement. I was ecstatic. Here was formal, articulate confirmation of my own political and racial views. And, more important, it represented an organized group of White people, and an organization, to which I myself could join.”*

-VE Veteran

*“…*[father’s] *racist attitudes towards Asians was so bad….at this restaurant…*[an Asian] *family came and sat next to us. It was just so bad I left because he is racist….I think he was probably very racist growing up, and I’m ashamed to tell you this even, but they always flew the Confederate flag at his parent’s house.”*

-Non-VE Veteran

Transmission of prejudice is the first factor and reflects narratives and networks. This is because at the root of violent ideologies are prejudicial attitudes toward others who are different from the self in terms of social categories such as race/ethnicity, gender/sexual orientation (e.g., Perliger, 2019). Moreover, prejudicial attitudes are learned early in life from families and close relationships such as friendships (Allport, 1954), and among veterans, continued exposure in the military and after military service (e.g., Curtin, 1997) suggests ideological messaging has a longer incubation period and stronger reinforcement given its occurrence in various contexts (e.g., civilian, military).

#### Trauma and adversity

*“…*[VE Civilian] *basically couldn’t cope with everyday life, couldn’t make ends meet, couldn’t pay the bills and didn’t know why he couldn’t do that. And someone told him that if he didn’t pay his federal taxes, if those taxes were left in his check, he could make ends meet. And then he started investigating that and someone told him that it wasn’t ratified properly in the Constitution, that it was illegal. And he went from there and got into anti-government, got into the militia…”*

-VE Civilian Family Informant

*“…the loss of a grandparent and a divorce is no reason to get involved in that* [radical group]*….It’s easy to bring somebody in that’s not doing well. Pat them on the back. Hey, you wanna talk about it? Hey, come here, man. Hey, why don’t you come out with me and the other guys….in the military, people that are not doing well and struggling personally, professionally, they are easy prey for anybody that wants to predate them for any reason”*

-VE Veteran Family Informant

*“…the CO said, ‘if you can find some way in the regs to get out, I suggest you do that’….and I found an article called Apathy. I didn’t exactly fit the bill, but I fit it well enough, and at that point, I was feeling pretty apathetic towards the military and so they gave me the general under honorable conditions….”*

-Non-VE Veteran

Trauma and adversity are the second factors, reflecting needs that could potentially inform grievances steering people toward radicalization and fueling VE. What is meant by trauma are adverse life events (e.g., childhood sexual abuse) that can potentially lead to a mental disorder (e.g., PTSD). Along with but distinct from trauma is adversity, defined as challenging life experiences (e.g., stressful job) that may not necessarily lead to clinical impairments. While adversity across the lifespan is a universal human experience, trauma can be a one-time experience or, in the case of compounded trauma, a series of experiences. Among veterans, military life experiences can be a source of trauma or adversity (e.g., combat, deployment) that, left unaddressed, can lead to greater postmilitary life challenges (e.g., illegal substance use).

#### Transitions

*“…more importantly this prompted me to type in the words ‘black on White crime’ into Google and I have never been the same since that day.”*

-VE Civilian

*“Serving in the military is not always easy and brings along many hardships and burdens; it changes people.”*

-VE Veteran Military Informant

*“I was brought in as an undesignated, no guarantees, no nothing….E1…I hated it. I was so angry…had I seen [the recruiter] I would gutted him…In retrospect, that was life-changing and character building…I no longer feel that way* [angry at recruiter]*….”*

-Non-VE Veteran

Transitions is the third factor and reflect narratives as well as needs that can become the basis for grievances. By transition, we mean situations or events that changes a person’s identity. From a criminological life course approach, these events are turning points in a person’s life that could be considered as usual (e.g., marriage) or unusual (e.g., crime) (see Sampson and Laub, 2017). As previously mentioned, military service, no matter how short or long, has a beginning and an ending. To a large extent, there is a predictable pattern inherent in the military lifecycle. Servicemembers start off as civilians, are transformed into warriors after joining the military, and become veterans as they transition back into civilian communities (Atuel and Castro, 2018b). But, even within the usual pattern of the military lifecycle, unusual events can still occur such as experiencing military indiscipline (e.g., being demoted) while on active duty, or homelessness during the postmilitary phase.

We note that while military service seems to mask the absence of civilian criminality, military laws operate and point to troubled behaviors within the military context. During postmilitary life when veterans are no longer governed by military rules and regulations, VE veterans either persist with old troubled behaviors or acquire new behaviors that increase their risk of coming into contact with law enforcement.

### The 3T model of military veteran extremism protective strategies

*“All around our society you see immoral behavior…lying, cheating, stealing, drug use and alcohol abuse, prejudice, and a lack of respect for human dignity and the law. In the not too distant future, each of you are going to be confronted with situations where you will have to deal straight-up with these issues. The question is…what will you do when that happens? What action will you take?…will you DO what you know is right? It takes moral courage to hold your ideals above yourself. It is the DEFINING aspect of your character. So, when the test of your character -of your moral courage comes -regardless of the noise and confusion around you, there will be a moment of inner silence in which you must decide what to do. Your character will be defined by your decision…and it is yours and yours alone to make.”*

-Retired General Charles Krulak (1997), 31st Commandant of the Marine Corps,

The 3T Model of Military Veteran Extremism Protective Strategies reflects three overarching approaches across the military lifecycle—Resisting Transmission of Prejudice, Addressing Trauma and Overcoming Adversity, and Navigating Transitions (see Table 7). Each general strategy share commonalities across the military lifecycle (e.g., intergroup contact for premilitary, military, postmilitary) and unique approaches within each phase (e.g., Finding the new you during the postmilitary phase), to which we now turn.

**Table 7:** 3T model (resisting transmission of prejudice, addressing trauma and overcoming adversity, navigating transitions) of military veteran extremism protective strategies^*^.

Stage	Transmission of prejudice	Addressing trauma and overcoming adversity	Navigating transitions
Premilitary	*Moral foundation* - Family resistance - Community norms - Wide-reader/Reading as a habit *Intergroup contact* - Friendships - Empathy	- Persistence - Emergence of the true self - Learning who you can trust	Setting expectations
Military	*Moral foundation* - Military values and norms (incentivized suppression of radical beliefs) - Courage (doing the right thing) *Intergroup contact* - Perspective taking - Openness to learning - Outgroup heterogeneity - Political and historical awareness of inequities	- Mental health and sobriety. In these instances, courage (doing the right thing) means asking for help.	*Pivoting* - Learn what it takes to get the job done *Understanding tradeoffs* - Choose what you can live with *Navigating military discipline* - You made a mistake. Learn, change, and move on. *Good mentor* - Find one *Separating military and civilian life* - Have one foot out the door.
Postmilitary	*Moral foundation* - Humanity (think bigger picture) - Justice (know the right thing) - Temperance (avoid extremes because they contain untruths or half-truths) *Intergroup contact* - Reconnect with old or make new friendships - Address inequities (speak up; do something)	- Mental health and sobriety programs at the VA (if you can find it). Military cultural competence matters. - Decompressing means destressing from military life.	*Finding the new you means:* - Revisit old dreams or pursue new ones. Dreams change. - Be Gumby if need be - Try or learn new things.

* Adopted from Table 4 of the Final Research Report for 2019-ZA-CX-0002.

#### Resisting transmission of prejudice

“*…the stoking of hatred was commonplace….While I was in the military and before the military, but the greatest impact was before the military.”*

-Premilitary Phase

*“Did I hear about people that were racist crap and stuff like that? Oh, yeah. It’s part of the system….When I was at* [name of installation]*, I did not participate but they blanket party a lady and I think if she’d been white, it would not have happened….”*

-Military Phase

“…[the plant managers] *would have their Dixie flag flying out in their front porch….Occasionally, they would query me as to if I had any interest in joining their cause….”*

-Postmilitary Phase

In spite of ideology exposure across the military lifecycle, the non-VE veteran sample resisted prejudice/hate, or influence stemming from radical/VE groups. And it was their moral foundation and intergroup contact that informed their cognitive or behavioral resistance.

##### Moral foundation

What is meant by moral foundation are fundamental notions of good or bad, or right or wrong that a person internalizes as part of their value system (Haidt, 2008). People’s moral foundations are initially shaped by the family, reshaped by the military, and can potentially be reinforced or recalibrated by the civilian transition experience.

Before military service, family values can influence resistance against prejudice (e.g., “*My mother was a hero of World War II…I am my mother’s son. I am sewn in my baby blankets anti-fascist, and anti whatever supremacist*…”). Also, community norms/members function as moral surrogates to reinforce family values (e.g., “….*we had a sense of community, where everybody whooped you. If they saw you doing something wrong, they would not wait and tell your mother. They spank you, and then tell your mother*)….”.

Also, reading as a childhood habit appears to be a protective factor for a variety of reasons. For some, books contain fictional characters displaying moral behaviors to be emulated (e.g., “[Louis Lamour books]…*as a kid, I focused on, hey, these guys do the right thing….they help anybody that needs help. They have values. They have integrity. They tell the truth….I think I’ve picked up a lot of my values from those books*…”). For others, books provide real-world exemplars of success (e.g., “*My parents subscribed to a book series about successful people and I read every book…it helped me focus*.”).

During military service, military values are inculcated, situating the servicemember to a level of identification directly tied to national ideals (e.g., “…*you take the oath of office and you raise your right hand and it says nothing about male or female, gay, straight, racial. It’s just you supporting the ideals of the constitution*…”). The superordinate military identity is a national identity that supersedes or subsumes diverse social categories (e.g., race, gender).

Moreover, the military identity is a value-based identity, whereby military values define servicemembers, in and out of uniform. Of all the military values, from antiquity to modern times, courage is the cornerstone (Castro, 2006). Courage is more than bravery or steadfastness in the battlefield (Moran, 2007), and can also mean doing the right thing regardless of the situation (Pigliucci, 2017). When applied to the transmission of prejudice, it means saying ‘no’ when an opportunity arises (e.g., “…*in my experience, people with mindsets like that aren’t interested in having a conversation. They’re interested in creating a conversion. I’m not willing to be converted. I know my values. I know who I am, and I know what’s right and wrong…It’s a decision that was made before the conversation happened*…”), or reporting someone to the proper authorities (e.g., “*I reported someone the other day for having a three-percenter bumper sticker on their car on base*…”).

After military service, it appears that courage continues to manifest in civilian communities (e.g., “*I stood between the proud boys and this* [homeless Vietnam Veteran] *on the ground and I said, what can we do to increase the peace in the community? Their answer to me was, you should join the proud boys because we are gonna secure the safety and peace of the community. My answer to them was, I’m now taking a picture of your license plate, and if you advance toward this man, you are gonna have to mess with a decorated Vietnam veteran….I will never give up helping veterans*…”).

Other values seem to be cultivated on the veteran’s own volition including justice (i.e., knowing the right thing, Pigliucci, 2017) and humanity (i.e., thinking the bigger picture, Peterson and Seligman, 2004). As described by a veteran, “…*more aware of the bigger picture and realized if you are going to have peace you have to have justice….which means there has to be social justice, so we are aware of things like Blacks Lives Matter, which we support*…”. Finally, there is moderation or avoidance of extremes (e.g., “*I think that’s how hate spread, with half truths. The truths that you can attach to and the untruths, since you do not know about it, you assume that’s true too….l I got educated on what these things people were talking about, I saw the half-truths….I do not go to extremes with the untruths*”).

Values are learned in many ways from different sources across the military lifecycle. During the premilitary and military stages, there was greater reliance on others to lay the moral foundation (e.g., family, military). Once set, veterans appear to cultivate other values on their own during the postmilitary phase. The point here is that the premilitary and military years till the moral soil and shapes the moral landscape for other moral values to flourish in the postmilitary years.

##### Intergroup contact

Meanwhile, intergroup contact involves exposure to and interactions toward different others in a variety of settings across the military lifecycle.

Before military service, family can be intentional about cross-cultural exposure that can include school and extra-curricular activities (e.g., “*Looking back in retrospect*, [mother] *did a really good job ensuring that we got the proper schooling, that we were exposed to different cultural things*”). In other cases, the community itself is culturally diverse, which can facilitate cross-cultural friendships (e.g., “*My best friend, I think, for a couple of years in elementary school was Colombian. Most of the kids in our neighborhood were Hispanic or Latino* …”). Interestingly, these early interracial friendships can come at a cost such as name-calling (e.g., “Then I was a n**** lover)….”.

During military service, the racial/ethnic diversity within the military itself becomes salient and appears to function as a forced stimulus (e.g., “…*in the military is when I was the first that I was really up close in quarters, living with, working with, eating with folks of other races from other places. It was definitely a learning experience to go from having this belief in diversity and equality to being just shoved right into a situation where it was what it was*”).

For some, military service provides the initial opportunity for cross-cultural exposure and contact, and one way to address prejudice in this situation is to adopt an openness to learning (e.g., “…*quite frankly, my first experience in dealing with people of other races, ethnicities…. I had experiences that I never would have had otherwise with other Americans from other backgrounds. It was really illuminating to deal with some of the Black soldiers who could be from more urban, well, for that matter rural environments. Learned a little bit about their lives. Not to mention Asian Americans in service*…”). Openness to learning can also occur when servicemembers are stationed overseas (e.g., “*I have a lot of experience that other people do not. I’ve lived in Africa, I was stationed there, and I probably do not have the same prejudices, but also those life experiences made a lot of difference*)”.

With greater contact comes outgroup heterogeneity or the awareness and recognition of individuated traits among different others (e.g., “…*basic training was I met the first, not the first Black person I’d ever met, but the first Black person that I actually admired, and he was my drill sergeant….He had wisdom. He knew when to push and he knew when to back off. He knew people, how to work with people*)”.

In other cases, perspective-taking and a growing awareness of political and historical inequities can stem from shared experience of historical events (e.g., “*One of the biggest, I guess, examples was George Floyd….For me it was really eye opening in that….Really there has not been a discussion and there was just no awareness of the different cultures and then the injustices that people face. It really is injustice….I think the injustice part I’m learning more about that and just seeing the inequality in new ways*”).

Interracial friendships can also develop and, similar to the premilitary phase, come at a cost which, in this instance, was disapproval from peers (e.g., “*One of the Black guys and I became friends….they got really upset about that. Why are you talking to the Black guy?*)”.

During the postmilitary phase, positive intergroup contact can occur in several contexts such as reconnecting with friends before military service (e.g., “…*then I renewed my friendship with* [names of friends], *and* [names of friends] *made sure I had everything I needed socially and financially and food wise. Then I got a job*…” a Veteran describing support of adoptive black people/persons/person family), or intentional cross-cultural learning after military service (e.g., “*I’ve learned more from the Native Americans in the last five, six years….learn more about the Native American Lakota way of understanding the earth and the way they see things and trying to reshape my thinking pattern more like theirs ‘cause theirs makes sense*”).

In the civilian workplace, positive interracial interactions can come at a cost of being ostracized by people in authority (e.g., “…*I feel privileged, that I was ever invited* [to lunch by Black coworkers], *but as a result of that, I was basically kicked off the plant by the plant manager who was in fact, a white supremacist…*[manager] *said, get the fuck out of my world, to be very crude about it*”).

Several strategies to counteract racism include calling it out (e.g., “*Now, when I hear it, I tend to take more action against it to speak up against it*”), or simple avoidance (e.g., “*I’d avoid the people that were acting that way, the racist people. I just stay away*…”).

Intergroup contact, especially between people of different races/ethnicities, is inevitable across the military lifecycle. With early research (Allport, 1954; Sherif, 1954/1988), mitigating prejudice across the military lifecycle includes the development of interracial friendships. Also, cross-cultural learning, which can begin with an open attitude while in the military, can be more intentional after military service. With greater positive contact, cognitive empathy toward different others or recognition of positive traits among different others, are possible building-blocks for postmilitary behavioral resistance that includes directly challenging racist comments, or avoidance of people perceived as racist.

#### Addressing trauma and overcoming adversity

*“….*[Mother] *was terminally ill by the age of 9 for me….taking care of her until the age of 12 when I was removed from the home because of her terminal illness….*[stepparent] *used the loving father trope….sexually assaulted me for two years….”*

-Premilitary Phase

*“There was no shortage of stress. There was no shortage of pressure.”*

-Military Phase

*“Once I got out, I went full on with* [drugs]*….Again it was all my own choice at the end of the day, those were just excuses that I used. Divorce and just depression, getting outta the military, losing my vocational, everything….”*

-Postmilitary Phase

Again, in spite of risk factors, these non-VE veterans demonstrated resilience by addressing their trauma and/or overcoming their adversity.

Before military service, persistence in overcoming adversity was found to be a critical trait that applied to various situations. In some cases, persistence was learned on one’s own to overcome challenges at school (e.g., “…*when we started writing cursor, I’m practicing, practicing.…taught me best I can so I took pride in my handwriting now because* [teacher’s name] *said I was from the devil* [for being left-handed]*”*), or during extra-curricular activities (e.g., “…*I was on the basketball team and people would harass me from the stands. I set a new record for the most free throws completed in a row that still stands at that school because I had to learn to ignore these idiots*…”). At times, persistence was modeled early on which, in time, became a life skill (e.g., *“*[Father] *just persisted in many things and that ended up being a theme through our lives and how we get through things…”*).

In challenging situations, people come to know a part of themselves or who they really are. In childhood, bullying situations appear to be the battleground whereby people came to experience the emergence of the true self (e.g., “*I’m a tall person…I was bullied quite a bit…I do not think it warped me. I’m a pretty secure person and, even though I was quiet and unsure in those days, I was still pretty secure*”; and, “*It bothered me to be bullied…most of that stuff, I just worked out on my own….I knew who I was…I developed that sense of self*”).

Sometimes, recognizing one’s own problem resulted in reaching out to someone who can help. In the case of childhood adversity, learning who to trust means seeking out a supportive person within one’s own community (e.g., “…*around the time I graduated from high school, I needed some counseling ‘cause I was starting to have some problems with my* [parent]*, and I saw a priest who has remained a lifelong friend*….”).

In the case of childhood trauma, help-seeking more likely occurs during the adult years, with assistance found in para-professional organizations (e.g., “….*It was shell shock my father had. They did not talk about it and this greatest generation, how they managed to survive with the shit they were carrying….My father became an alcoholic and it got worse as he aged. I’m an ACOA* [adult child of an alcoholic]…*I’ve been through some counseling….I’m sure I went into the military with PTSD*”). In other cases, (compounded) trauma results in a clinical diagnosis requiring professional clinical services (e.g., “*My current psychiatric diagnoses which go back are complex PTSD…. my doctors have told me…unfortunately you are too wounded*…”).

During military service, mandated services appear to be the norm rather than voluntary help-seeking (e.g., “*We went on deployment…we got high, it was PCP. When we got back they tested us….they sent me through drug and alcohol treatment*…”).

After military service, mental health services were sought for different reasons. Foremost is marital counseling, which appears to be a gateway for future counseling services. This trend was observed among those who went to a VA facility (e.g., “…*I wish that I’d gotten counseling earlier because….that poor* [ex-spouse]….*I could have done a hell of a lot better….I have been going counseling for the past 30 years…That’s through the VA.…I did go to counseling for myself, for anger management. I went to parenting counseling and it was good that I did*”), or a civilian provider (e.g., “*When I first sought out counseling….one of the questions was asked….‘what are you feeling?’ I realized that I did not have a clue….in retrospect I see that’s a massive red flag….that was all they needed to know, basically about me, that here’s a guy who needs some help over here….started out to be marriage counseling with* [ex-spouse]*….After the marriage, I committed talking to a therapist….I was able to have a real epiphany….become much more in touch with myself as a person. Much, much, much more happy*”).

However, seeking help from a VA (vs civilian) facility seems more appropriate for military-related issues such as disability (e.g., “*I was recently granted 100 percent service connection….it took me 21 years, but as Steve McQueen said floating on the coconut bag in the movie Papillion, ‘I’m still here you bastards’*”), or even discharge status (e.g., “[from general discharge to honorable]*…a VA guy…did all the groundwork and got me a copy of this new discharge thing.* [VA guy] *was appalled that I had this thing and that I had met some prejudice along the way”*).

Military culturally competent care in the VA is further underscored when receiving clinical services. As one veteran shared of their experience with a civilian therapist, *“…no understanding of veterans….rated me as impulsive, having a death wish, as a violent person, and it was – I have no idea where* [therapist] *got what* [therapist] *got. I’ve never committed an act of violence, even on active duty.”* When this same veteran connected with the VA, they described the experience as *“…the VA is what turned things around for me. I was able to get connected with the SARP* [Substance Abuse and Rehabilitation Program] *Program.”*

In addition to seeking mental health services, some expressed decompressing as destressing from military life (e.g., “*I went through some pretty rough times and I stayed afloat. I do not have the stressors in my life anymore and you do not realize how stressful that military is…decompressing. That took a while. Took a couple of years to totally be out of it and decompress and stop.”*).

At times, destressing occurs within a supportive network and in pursuit of a new purpose (e.g., *“My mental health, from my PTSD from the military, I think is helped by my social justice activism. I focus on other people’s problems more than my own…. My kids help me with my mental health and being a father helps me with my mental health and my social justice helps me with my mental health and my stress from the military”*).

People experienced adversity across the military lifecycle. Early on, acquiring certain life skills can prove to be useful over time. When one leaves the military, gaining and appreciating a new rhythm opposite to the tempo of military life, or finding a new purpose in the civilian world are strategies to overcome military-related stress. Regardless of where one is in the military lifecycle, personal challenges are overcome with the presence of a social network that is a source of emotional, moral, and/or social support.

However, for trauma, especially when left unaddressed, there is the increased potential of developing clinical disorder(s) that can escalate into compounded trauma stemming from childhood (e.g., abuse) and military (e.g., deployment) experiences. This means seeking out appropriate healthcare services found within the military health system and when applicable, continuing into the veteran health system (VA). The importance of VA services cannot be understated because military cultural competence matters. Simply defined, military cultural competence “pertains to a provider’s attitudinal competence, cognitive competence, and behavioral competence in working with servicemembers and veterans” (Atuel and Castro, 2018a, p.77). Admittedly, one of the significant barriers to help-seeking is the stigma around mental illness, especially while a servicemember is on active duty, yet it needs to be overcome before therapeutic services are rendered. Hence, VA access becomes more imperative in addressing mental health issues. Access, though, is not synonymous to successful engagement, and successful engagement is not a one-time visit. For some veterans, it is safe to assume that successful engagement with the VA is an adversity in and of itself, a challenge that can be overcome with persistence.

#### Navigating transitions

*“….when I graduated from HS, I did not have any ambition to do anything, but my folks were not gonna let me sit on the couch. I did not wanna get a job…go to school. I figured, I’ll join the Army….my Dad was a reserve officer in the Army….”*

-Premilitary Phase

*“They flagged me ‘cause I was overweight….My automatic promotions stopped immediately.”*

-Military Phase

*“…*[when 20-month old child died] *I just lost it. That’s when I went to Skid Row….I became homeless…I just gave up….”*

-Postmilitary Phase

Yet again, in spite of risk factors, these non-VE veterans demonstrated resilience in navigating their various transitions across the military lifecycle.

Before military service, some people set expectations for military service such as pursuing educational opportunities (e.g., *“Well, primarily it was I needed to go to college”*), or pursuing one’s ideals (e.g., *“…I joined the military to organize against the war…”*). In other cases, enlistment was to escape from an adverse situation (e.g., *“I needed to get outta my city cause was my city was rough, and I did not wanna die on purpose or by accident”*), or simply the last option at that time (e.g., *“I did not join the military out of patriotism. It was really the last option for me. Patriotism and that national pride developed afterwards. I say national pride, but I wanna be very clear, not nationalist”*).

During military service, people managed military life by engaging in several strategies to keep up with the tempo of military life. By and large, these were cognitive/behavioral approaches to navigate military life in alignment with military values and culture.

The first strategy is pivoting or learning what it takes to get the job done. Pivoting can reflect thoughts or actions in response to direct military orders (e.g., *“…they made me a mail clerk….I learned how to forward mail….box mail….sort mail….change mailbox combinations. I learned how to do everything but accountable mail…*), military educational offer (e.g., *“…scholarship was for something else, so that made me pivot…”*), or personal career choices (e.g., *“For a long time, I guess, there were two things that I had considered career wise. One was to be a doctor and the other I really wanted to be* [name of elite operations]*…and that was not gonna be possible….my focus shifted to – I needed a career that I felt as a* [parent] *and* [spouse] *could put food on the table…so I just transitioned to* [healthcare occupation]*…”*).

Related to but distinct from pivoting is understanding tradeoffs or choosing what you can live with (e.g., *“…two of us were in 2^nd^ place trying to choose the 2^nd^ best option, everything after that was junk. How do you resolve this? Well, I looked at the options and realized that some of these bad options were for very short periods of time….I’ll take one of these 1 year tours. I chose Turkey”*).

Like everyone else, servicemembers commit errors, professional or personally. When this occurs, navigating military discipline may entail recognizing that one has made a mistake, learning from that mistake, and/or moving past that mistake (e.g., *“Those things happened, and there was some difficult breakups and difficult things, but for the most part, I saw my part in it. Either just accepted and moved on, and made the changes and moved on…”*). Interestingly, military discipline could turn out to have positive unintended consequences (e.g., *“…I can tell you that I did clerical work for a while, I was disciplined at one point and they made me a male clerk, which was fine, I kinda liked that*”).

Another strategy is to find good mentors, which the chain of command within the military provides ample opportunities for servicemembers to be guided by others who can help navigate life, military-specific or in general (e.g., “*Early on, a particular officer pulled me aside because I was struggling. He said, I remember to this day, he said, ‘You cannot hide a goat in a flock of sheep. People are gonna see you for who you are, and people are gonna see them for who they are. It might take some time, but they are gonna be seen. Everybody is seen for who and what they are’. That has stuck with me my entire life*”).

Because military service comes to an end, another strategy is to have one foot out the door. This could mean delineating space between military and civilian life (e.g., “*I refused to go to military housing…I do not wanna live with military people while I’m not in the military space. I wanted to close the door on that, my 9 am to 5 pm…and have my civilian life*…”). Or, when the time to separate from military service is fast approaching, having a plan in place makes the transition something to look forward to (e.g., “…*I have a job lined up…I wanna see if I can translate all of my military experience, see what I’ve learned and see if I can get in and see if I can make a difference….I’m looking forward to my new work and perhaps a new social group*….”).

After military service, finding the new you is perhaps one of the most pressing and enduring challenges of the civilian transition. While this ‘new you’ could pertain to the self as situated in a new job, new neighborhood, or with new friends, above and beyond basic needs (e.g., housing) was the pursuit of an identity that provided meaning and purpose to life.

One strategy involved dreams or aspirations, which meant revisiting old dreams/pursuing new ones (e.g., “*My interest had shifted a little bit, so I dropped out of art school….got a BA in psychology….worked as a* [VA] *social worker*. *During that time, I took some evening classes in art….one of ‘em was a pottery class, and I just got hooked….Then, using the GI Bill money, I quit my job….started selling pottery*……”). The point here is people acknowledged and allowed their dreams to change (e.g., “*I wanted to be a doctor my whole* [military] *career….I did all my pre-med….I couldn’t get past organic chemistry because I think my PTSD, I couldn’t get past organic chemistry. I’ve taken the tests to go to medical school, and I didn’t do well on them….I came out of the military and spent my time since then, social justice. It’s a whole new me and whole new purpose*….”).

Pursuing a new sense of self also means being gumby if need be or being flexible. For the most part, this strategy was observed during the early stages of the civilian transition when people did not have concrete plans as of yet or just trying to get their bearings back in the civilian world (e.g., “…*Then I got a job…fixing Xerox machines….Then I felt like it was time to move on…Then I found a job which required me to live in, it was a singles hotel, and so I kinda went from there….worked for the* [name of newspaper]*…went back to* [midwestern state] *where I could go to school for free*…”).

Related to this approach is trying or learning new things (e.g., “…*I used my GI Bill to get my first degree……I could dumpster dive for food…I still dumpster dive once in awhile just to stay in practice*…”), which can have positive associated effects in that one acquires new skills, professionally and socially (e.g., “… *have gained, what do they call it? It’s like social IQ…it’s that social ability to work with people, ability to get along with people. That has grown very much since I left the military ‘cause it doesn’t happen in the military. I don’t think*”).

With greater engagement in civilian life, comes an enlargement of the sense of self reflecting both the military and non-military identities (e.g., “*I am coming to a place where my military identity is an important part of my life, but it’s no longer an identity. My identity now is as a* [profession 1], *as a* [parent]*, as a* [profession 2]*. I can count being a veteran as part of who I am, but not who I am. The release of that identity is actually making it easier for me to get through*….”). The military identity, which is still part of the overall self-concept, can become salient though under certain conditions (e.g., “*I don’t tell them I’m a veteran until it comes up, and we just do the job. I don’t lead with my service….I am a veteran and if you’re messing and screwing over veterans and they’re homeless and their mental health and all that stuff, you’re gonna hear from me. I don’t lead with it, but I own it*”).

While the military identity was critical within the military environment, navigating civilian life after military service will require other ways of defining the self *in addition* to the military identity. The military identity is not a lost identity, but co-exists with other identities that acquire more importance during the civilian transition process (Atuel and Castro, 2018b). Just as military identity dictated military life, the military identity takes on relative importance after military service and can exist side-by-side with new ways of describing the self. This was the case for our sample who were able to find new anchors, broadening who they were above and beyond their military identity.

Navigating transitions pertains to learning and adopting a set of values, attitudes, and behaviors normative to civilian life, military life, and veteran life. It is also about setting and managing expectations across the military lifecycle. Owing to the predictability of the military lifecycle, transitions can be planned, to a degree. Owing to the unpredictability of life, in general, it appears people acquire a degree of flexibility toward accommodating changes in their life.

As a related consequence, the sense of self is broadened through the accumulation of diverse experiences. What emerged are open-minded veterans (Atuel and Castro, 2018b): while the military identity continues to define the self, it is relegated as part and parcel of the constellation of identities that enable veterans to thrive and flourish in their respective roles and professions as civilians.

## Concluding remarks

*“But above all we should decide who we want to be, what we want to be like, and what way of life we want to lead. This is the toughest of all our deliberations…nevertheless, whether it is by a kind of good luck or by innate goodness or by parental training, some do pursue the right path through life…the decision comes down entirely to each person’s individual nature….”*

-Cicero (2008), *On Obligations*, 1.117,119

Thus far, we have found individual-level strategies that steer veterans away from the radicalization pathway leading up to VE. What then can be done at the group-level?

### Strengthening the civilian-military collaboration

*“…catch ‘em I think when they are young. It takes proper education in a lot of things….”*

In the aftermath of J6, Retired General Peter Chiarelli reasoned that the military represents a cross-section of American society where all forms of extremism thrives, “….we get these people when they are 18, sometimes older, so we are battling to change all the prejudices and wrong things they have learned up until they put on a uniform” (Fanz, 2021). Prejudice is, foremost, a civilian community problem. And who exactly is the civilian community? Here we invoke Allport (1954) and look to families, schools, and places of worship and, more importantly, place the greater burden of intercultural education on the school to “set before the child a higher code than is learned at home” (p. 511). Indeed, the field of intercultural education (e.g., Coulby, 2006) has evolved and continues to seek multidisciplinary best practices (e.g., Holliday, 2018) to address the changing cultural and global dynamics (e.g., migration patterns) that affect schools (e.g., increase in migrant children).

Meanwhile, U.S. public schools and libraries are currently being challenged with a book ban and textbook censorship movement. Given that habitual reading before military service was identified as a protective factor, it is not far-fetched to imagine that a consequence of the censorship movement is the raising of a generation of K–12 students with a narrower view (at best) or no view (at worst) of intercultural education. Obviously, from this pool will come the next cadre of servicemembers who will comprise the military force.

Relatedly, the current state of affairs in the military is to turn a blind eye to the extremism problem. But, prejudice is and will continue to be a challenge to the military, especially if little is done at the civilian/premilitary phase. Elsewhere, we argued that as the intermediary status between civilian and veteran, the military is the most potent influencer of change (Atuel and Castro, 2024). The military is a value-based institution that has the power and authority to shape and reshape norms, values, and identities. It is at the crux of instilling codes of honor and duty (e.g., Castro, 2006) above and beyond military service.

Instead of taking a siloed approach to addressing the issue, there needs to be a concerted effort among different communities, especially between the school and the military, to work together. What is at stake is the moral foundation, which is a work in progress, where values can and should be reinforced across the military lifecycle. This is a long-term investment that needs to be made across institutions.

### Creating a military culturally competent workforce

*“Mental health is an issue, and I wish that I’d gotten counseling earlier….”*

Some veterans experience trauma before military service (e.g., abuse) and/or stemming from military experiences (e.g., deployment). Regardless when trauma occurred, it needs to be addressed. A recent review (see Barr et al., 2022) identified clinical (e.g., Cognitive Processing Therapy, Resick et al., 2016) and non-clinical approaches (e.g., Moral Injury Reconciliation Therapy, Lee, 2018; see Koenig and Al Zaben, 2021) used to treat trauma, most of which are available in the Department of Defense Military Health System (2020) or Veterans Affairs Hospitals.

In an all-volunteer force, there are fewer servicemembers and veterans in our midst compared to previous wars and conflicts. This civilian-military divide is more than numerical, it is a cultural gap one as well. Hence, behavioral health providers within the military, veteran, and civilian health settings need to develop military cultural competence, defined as a provider’s attitudinal (e.g., beliefs about the military), cognitive (e.g., knowledge of military culture), and behavioral (e.g., skill set) competence in working with servicemembers and veterans (Atuel and Castro, 2018a). There is no better time for behavioral health providers to expand their toolkit to include assessing and monitoring for radicalization and VE. Short term, civilian-based threat assessments for extremism being used in operational, correctional, or forensic mental health (Logan and Lloyd, 2019; see Scarcella et al., 2016 for a review) could be adopted. The long-term goal will be measurement development and implementation within systems of care for the military and veteran populations.

### Forging military-veteran mentorships

*“….if you joined the military, immediately join a veterans organization, immediately, and a veteran organization that is focused on making sure that whatever happens to you in the military is addressed properly….there should actually be national legislation that requires any member of the military to be involved in a veteran’s active duty support organization right off the top, not after, but right off the top”*

As mentioned, there is a predictability to the military lifecycle with military service having a discharge date. With this end in mind (not sight), it seems prudent for servicemembers to begin the transition process early on by connecting with a veteran group. A recent review (Mercier et al., 2023) found that veteran peer support groups can potentially positively influence the well-being of veterans, servicemembers, and their families, particularly in the areas of health, life skills, and social integration (p. 11). Specifically, informal support groups (e.g., breakfast clubs) function to provide mutual support of shared military service and civilian transition experiences (e.g., McDermott, 2021).

It is never too early to plan for transition. For starters, establishing veteran connections while still on active duty could be seen as a type of mentoring. Earlier, we reported on mentoring opportunities within the military chain of command as a protective factor against radicalization and VE. Now we are advocating for a veteran-active duty mentoring to proactively address transition challenges. A related issue is these mentoring interactions need to be conducted in a group. Generally, the civilian transition process is a solitary experience (Atuel and Castro, 2018b). For while the military identity was formed in a group, the veteran identity, for the most part is an individual activity. Hence, veteran groups can function as new anchors of identity, values, and a sense of purpose. This mentoring across transition phases is a long-term agenda for the military and veteran communities to invest in. Short-term, we recommend vetting different veteran groups for inclusion in the Transition Assistance Program as resources for informal support.

### Directions for future research and limitations of the present study

Future avenues pertain to the limitations of the present study. The sample size of this qualitative study is small. Future research should replicate with a larger sample size that is recruited from a more representative pool of the veteran population (e.g., in collaboration with the VA) to attenuate the sampling bias of the snowball and online recruitment methods used in the study. Another limitation is the inclusion of open-source data that could be a source of measurement error; hence, future research should focus solely on primary data collection. Nevertheless, the results of the present study offer insights into individual-level factors that motivate and situate veterans to outright resist hate, prejudice, or violent ideologies.

Future research should also reflect the full spectrum of radicalization and VE, with comparison groups that include veterans who hold radical/VE beliefs privately, but do not act on them (cognitive radicals), veterans who provide instrumental/financial support to radical/VE groups, but not part of them (supportive radical), veterans who join radical/VE groups but do not act violently (non-violent radical), and veterans who used to belong to radical/VE groups and later denounced the group (former radicals).

## Data Availability

The datasets presented in this study can be found in online repositories. The names of the repository/repositories and accession number(s) can be found below: Quantitative variables are part of the datasets submitted to the National Archives of Criminal Justice Data (NACJD_NIJ-194832).
